# Health Care Professional–Supported Co-Design of a Mime Therapy–Based Serious Game for Facial Rehabilitation

**DOI:** 10.2196/52661

**Published:** 2024-01-24

**Authors:** Daniel Lima Sousa, Silmar Teixeira, José Everton Fontenele, Renato Mendes Santos, Leynilson Pereira, Rodrigo Baluz, Victor Hugo Bastos, Ariel Soares Teles

**Affiliations:** 1 Parnaíba Delta Federal University Parnaíba Brazil; 2 State University of Piauí, Campus Parnaíba Parnaíba Brazil; 3 Federal Institute of Maranhão, Campus Araioses Araioses Brazil

**Keywords:** serious game, serious games, facial recognition, face estimation, computer vision, facial rehabilitation, face, rehabilitation, physiotherapy, mime therapy, co-design, human face estimation, facial palsy, facial paralysis, motor rehabilitation, exergame, physiotherapists, psychologists, participation

## Abstract

This research letter presents the co-design process for RG4Face, a mime therapy–based serious game that uses computer vision for human facial movement recognition and estimation to help health care professionals and patients in the facial rehabilitation process.

## Introduction

Facial paralysis is a consequence of damage or injury to the facial nerve, resulting in functional impairments. A challenge of rehabilitation through exercise repetition is maintaining patients' engagement and motivation in the intensive and repetitive execution of the exercises necessary for successful rehabilitation [1]. Repetitive and intensive movements are recommended for progress in treatment [2], and the variety of movements has significant effects on patient recovery [3].

In motor rehabilitation, exergames—serious games that require physical exercise to play—add fun to exercises and allow patients to forget about their condition and focus on the game [4]. Studies conducted with games for motor rehabilitation have achieved promising results [5] on patient motivation and engagement [4]. This study aimed to co-design *RG4Face*—an exergame for facial rehabilitation.

## Methods

### Ethical Considerations

This study was approved by the Research Ethics Committee of Universidade Federal do Delta do Parnaíba (5.632.311). The first author (DLS) provided explicit consent for use of his image in Multimedia Appendices 1 and 2.

### Study Design

**Figure 1:**
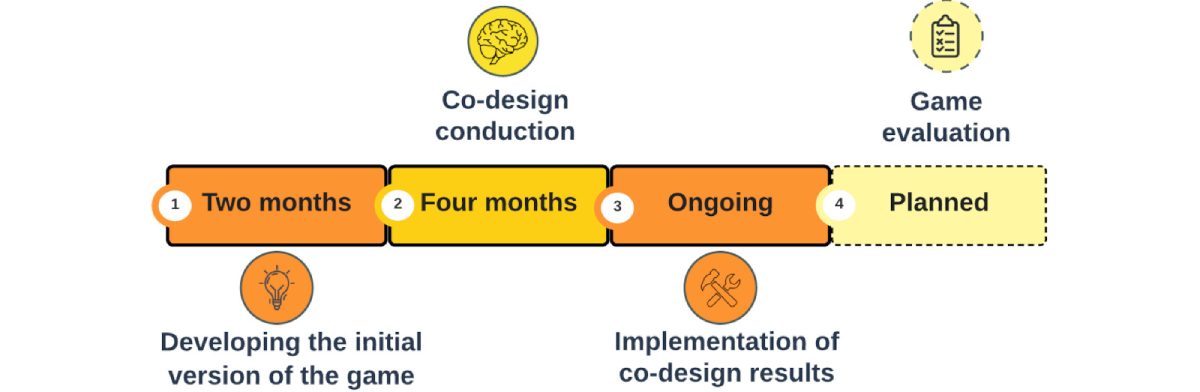
Co-design timeline.

To develop *RG4Face*, a co-design procedure (Figure 1) was conducted with physiotherapists (n=16) and psychologists (n=5; Multimedia Appendix 3) to obtain the necessary knowledge on the game requirements.

In the first stage, a version of the game was developed with an initial idea (Multimedia Appendix 1). In the second, we recruited physiotherapists and psychologists to participate in co-design meetings (August to November 2022) and answer a questionnaire. We then presented the game to the participants and allowed them to make suggestions. The prototype was essential to encouraging participation during meetings. In total, 5 meetings were held—4 with physiotherapists and 1 with psychologists. The main activities of the meetings were brainstorming sessions, in which the generation of game requirements was encouraged for their incorporation into visual elements, gamification, and game mechanics. Meeting results allowed for the creation of a list of requirements. As a third stage, we are concluding the implementation of *RG4Face* based on the produced requirements. The game code was implemented in JavaScript to provide new features for facial rehabilitation via the Rehabilite Game platform [6].

## Results

Per its initial conception, *RG4Face* uses computer vision (via a camera) for capturing, recognizing, and estimating human facial movements. The game prototype was implemented via the MediaPipe face mesh [7] to enable the recognition and use of 1 movement (eg, raising eyebrows; ie, frontal muscle) to control game elements. The game involves a spaceship moving horizontally across the bottom of the captured video window and firing a projectile when face movement is detected. The main objective is to hit triangles that randomly appear on the player's face.

Table 1 presents participants’ suggestions during co-design, game requirements, and rationales.

*RG4Face* is in the testing phase and, prior to evaluations, can recognize 6 movements used in mime therapy to improve facial muscle strength and mobility (Multimedia Appendix 2). To implement the recognition of these movements, MediaPipe was used [7]. The face mesh model allows for the real-time tracking of 468 3D landmarks on the human face that represent important facial features (eg, eyes, eyebrows, nose, and mouth). Distances between landmarks are calculated to recognize movements.

*RG4Face* provides a mirror therapy feature [8], which can mirror the healthy side of the face to create a visual illusion that can help reduce pain and improve function. *RG4Face* allows for parameter adjustment on the Rehabilite Game platform. Health care professionals can choose specific game mechanics for each rehabilitation case, thereby customizing the game according to patients’ needs and difficulties.

**Table 1:** Functional and nonfunctional game requirements from the co-design procedure.

Participants’ suggestions	Refined requirement	Rationale
Improve the game scenario Improve the representation of the ship and projectiles Choose attractive colors and contrasts	Improve game colors and elements: border, ship, projectiles, and collision	Enable the game to become more attractive and stimulating
Include levels with difficulty levels	Provide difficulty levels	Gamification for each level, depending on the patient's condition
Provide an option of mirror therapy for the game	Implement a mirror therapy simulation	Patients with Bell palsy can benefit from it
Implement better game mechanics for rewards Promote progression in the game	Create a scoring and bonus system	Increase patients’ adherence to and engagement with treatment
Movement sensitivity must be customized according to the patient's degree of disability	Implementation of sensitivity levels for motion recognition	The level of sensitivity respects the movement capacity of each patient
Create metrics on the game platform to monitor the rehabilitation process	Provide in-game metrics	They are interesting for the health care professional to follow the patient's progress
To avoid causing botheration to some types of patients, the sound should be optional	Allow game sound to be optional (ie, turn off the sound)	The sound may be unnecessary for some patients
Consider visual acuity of the players The game scenario should be full screen	Make game screen full, automatically adjusting to the aspect ratio	Game elements should be clearly visible
Head movement should not influence the game Calibration is essential to avoid false positives and false negatives of movements	Perform a prior calibration of the player’s face	Adjustment of the distance between player’s face and screen, in addition to improving movement recognition

## Discussion

We co-designed a serious game for facial rehabilitation that represents a potential new approach to improving patients’ adherence to facial rehabilitation. The co-design procedure allowed stakeholders to participate in defining game requirements, thereby empowering the tool to meet the needs and expectations of patients and be more engaging and motivating.

Although there are studies that focus on games for rehabilitating specific parts of the face (eg, eyes [9] and mouth [10]), to our knowledge, no serious game for facial rehabilitation has been proposed that can recognize the face movements used in mime therapy. This study proposes the first such exergame.

Our results demonstrate that the co-design approach was effective for creating a serious game with the potential to meet patients' needs. We plan to evaluate the game with health care professionals, healthy participants, and patients with facial paralysis.

## Appendix

Multimedia Appendix 1 Video presentation with the game prototype before the co-design procedure.

Multimedia Appendix 2 Video presentation with the game after implementing requirements from the co-design procedure.

Multimedia Appendix 3 Demographic characteristics of participants.
