# Supplementary material for: Health Care Professional–Supported Co-Design of a Mime Therapy–Based Serious Game for Facial Rehabilitation
Source: JMIR Serious Games. 2024 Jan 24;12:e52661. doi: 10.2196/52661 (PMC10851117; doi:10.2196/52661)
Supplement: Multimedia Appendix 3 [file games_v12i1e52661_app3.docx]

Table 1. Physiotherapists.

|  | **N** | **%** |
| --- | --- | --- |
| **Gender**  Male  Female | 4  12 | 25  75 |
| **Age**  18-29  30-39  40-49  50-59 | 7  7  1  1 | 43.75  43.75  6.25  6.25 |
| **Time since graduation**  ≤ 5 years  > 5 years | 7  9 | 43.75  56.25 |
| **Time of experience**  ≥ 5 years  < 5 years | 10  6 | 62.5  37.5 |
| **Have you ever treated patients with facial paralysis?**  Yes  No | 14  2 | 87.5  12.5 |
| **Have you ever used a computational tool (e.g., a game) in motor rehabilitation of patients?**  Yes  No | 2  14 | 12.5  87.5 |
| **Would you use games to help motor rehabilitation of patients?**  Yes  No | 16  0 | 100  0 |
| **Total** | **16** | **100** |

Table 2. Psychologists.

|  | **N** | **%** |
| --- | --- | --- |
| **Gender**  Male  Female | 2  3 | 40  60 |
| **Age**  30-39  40-49 | 3  2 | 60  40 |
| **Time since graduation**  ≤ 5 years  > 5 years | 0  5 | 0  100 |
| **Time of experience**  ≥ 5 years  < 5 years | 5  0 | 100  0 |
| **What is your approach to studies and work?**  Cognitive Behavioral Therapy  Person-Centered Humanistic Therapy | 2  3 | 40  60 |
| **Total** | **5** | **100** |
